# Cardiac Surgery Management in Patients With Liver Cirrhosis

**DOI:** 10.31083/RCM49476

**Published:** 2026-08-18

**Authors:** Adam Ryszard Kowalówka, Mikołaj Jodłowski, Aleksandra Kiszka, Ryszard Bachowski, Radosław Gocoł

**Affiliations:** ^1^Department of Cardiac Surgery, Upper-Silesian Heart Center, 40-635 Katowice, Poland; ^2^Department of Cardiac Surgery, Medical University of Silesia, 40-635 Katowice, Poland

**Keywords:** cardiac surgery, liver cirrhosis, hepatic cirrhosis, Model for End-Stage Liver Disease, Child–Pugh, VOCAL-Penn score, perioperative management, cardiopulmonary bypass

## Abstract

Liver cirrhosis substantially increases perioperative morbidity and mortality in patients undergoing cardiac surgery through coagulopathy, portal hypertension, cirrhotic cardiomyopathy, renal dysfunction, and immune dysregulation. Conventional cardiac risk scores underestimate this risk because these metrics do not incorporate variables related to hepatic function, highlighting the need to integrate the Child–Turcotte–Pugh (CTP) and Model for End-Stage Liver Disease (MELD) scores. Novel predictive models, such as the Veterans Outcomes and Costs Associated with Liver Disease – Penn (VOCAL-Penn) and Mayo Postoperative Mortality Risk Score (Mayo scores), demonstrate superior discriminatory performance by incorporating surgery-specific variables. This narrative review synthesizes the evidence on risk stratification, preoperative optimization, intraoperative management, and postoperative care in patients with liver cirrhosis undergoing cardiac surgery. A structured, multidisciplinary approach that incorporates systematic hepatic assessment, careful patient selection using integrated cardiac-hepatic risk models, tailored intraoperative techniques to minimize cardiopulmonary bypass (CPB)-related injury, and vigilant postoperative organ support can improve outcomes in this high-risk population. This review provides practical, phase-oriented recommendations with evidence grading, critical discussion of controversies, and acknowledgment of the limitations inherent in the available literature.

## 1. Introduction

Chronic liver disease (CLD), including cirrhosis, represents an increasing public health burden in aging populations where classical cardiovascular risk factors such as arterial hypertension, diabetes, obesity, and smoking frequently coexist [1]. Advances in hepatology and the development of antiviral therapies have improved survival outcomes in patients with CLD and, consequently, have expanded the population of cirrhotic patients presenting for cardiac surgical intervention due to significant coronary artery disease or structural valvular pathology requiring surgical correction [1]. This review focuses specifically on patients with established liver cirrhosis undergoing cardiac surgery. Meanwhile, early-stage CLD without cirrhosis, non-cirrhotic portal hypertension, and acute liver failure are beyond the scope of this review. This epidemiological shift has created an unprecedented clinical challenge for cardiac surgeons, as cirrhosis confers a markedly elevated perioperative risk profile, with an estimated 4–7 fold increase in mortality compared with non‑cirrhotic populations; however, liver dysfunction remains conspicuously absent from widely used risk stratification models, including European System for Cardiac Operative Risk Evaluation II (EuroSCORE II) and the Society of Thoracic Surgeons (STS) calculator [2,3,4,5]. The pathophysiology underlying this elevated risk is multifactorial and complex. Cirrhosis induces a characteristic circulatory phenotype defined by a hyperdynamic circulation with reduced systemic vascular resistance, latent cirrhotic cardiomyopathy with impaired contractile reserve that becomes unmasked during acute hemodynamic stress, and a rebalanced but fundamentally fragile coagulation system characterized by simultaneous bleeding and thrombotic potential [6,7]. Additionally, cirrhotic patients experience functional renal vasoconstriction despite normal renal perfusion pressure and profound immune dysregulation that predisposes the patients to serious bacterial infections and sepsis. Cardiopulmonary bypass (CPB) amplifies these underlying abnormalities through massive inflammatory activation, progressive hemodilution-induced coagulopathy, and hepatic ischemia-reperfusion injury [8]. Consequently, simple extrapolation of cardiac risk assessment strategies and perioperative management protocols from the general cardiac surgical population to cirrhotic patients is clinically inappropriate and potentially hazardous. Emerging evidence increasingly supports an integrated, multidisciplinary “heart–liver team” approach that strategically combines conventional cardiac risk models with hepatology-specific assessment tools, including the Child–Turcotte–Pugh (CTP) score, Model for End-Stage Liver Disease (MELD), MELD-sodium (Na), and novel predictive models such as Veterans Outcomes and Costs Associated with Liver Disease – Penn (VOCAL-Penn) and Mayo scores. This comprehensive approach enables more accurate risk stratification, guides patient selection for surgical versus transcatheter or medical management, and informs tailored perioperative optimization strategies [4,5]. The present review synthesizes contemporary evidence to provide a structured, phase-oriented practical algorithm for the perioperative management of cirrhotic patients undergoing cardiac surgery, encompassing preoperative risk assessment and systematic optimization, intraoperative anesthetic and hemodynamic strategies, CPB management with emphasis on minimizing tissue injury, and vigilant postoperative care with early detection of organ-specific complications.

## 2. Literature Review

### 2.1 Risk Stratification: Before Surgery Begins

#### 2.1.1 EuroSCORE II and STS

EuroSCORE II and STS calculators are widely used but systematically underestimate risk in cirrhotic patients [2,3,4,5]. A patient classified as “low-risk” by standard scores frequently experiences mortality rates of 10%–20% because these models do not include variables reflecting liver synthetic function (albumin, bilirubin, international normalized ratio (INR)), portal hypertension, or renal status in the context of cirrhosis [4,5]. Consequently, dual risk assessment is recommended for every cirrhotic patient: a standard cardiac score plus liver-specific tools such as CTP and MELD [4,9]. The failure of conventional cardiac risk models in cirrhotic populations stems from fundamental physiological differences. Cirrhotic patients exhibit altered pharmacokinetics, impaired coagulation reserves despite apparently “normal” laboratory values, and hemodynamic instability that becomes more pronounced during surgical stress [6,7]. Additionally, renal function in cirrhosis is highly vulnerable to perioperative hypotension and reduced cardiac output, yet traditional models do not account for hepatorenal axis dysfunction [10]. Furthermore, portal hypertension creates unique vascular considerations that affect both bleeding risk and hepatic perfusion, which standard scores completely overlook. The presence of varices, ascites, and encephalopathy, cardinal features of decompensation, is not captured by EuroSCORE II and STS calculations, despite the profound influence of decompensation on postoperative outcomes and complications [4,5]. Therefore, integrating liver-specific assessment tools, including CTP, MELD, and MELD-Na, into the preoperative evaluation is essential for appropriate patient selection and risk counseling [4,9,11].

#### 2.1.2 Child–Turcotte–Pugh Classification

The CTP classification profoundly influences perioperative outcomes and complication rates in cardiac surgery [4,5,12]. Patients with CTP A demonstrate acceptable perioperative mortality (8%–15%) with morbidity related primarily to standard cardiac surgery risks, rather than hepatic decompensation [4,12]. Patients with CTP B face substantially higher mortality (20%–50%), along with increased rates of acute kidney injury, sepsis, hepatic decompensation, and prolonged intensive care unit (ICU) stays [4,5,10,13]. Patients with CTP C experience prohibitive mortality (~70%) due to severe coagulopathy, refractory hypotension requiring massive vasopressor support, fulminant hepatic failure, and multiorgan dysfunction [6,7,12,13]. In addition to mortality, CTP status predicts patterns of postoperative morbidity [4,5,12]. Higher CTP scores correlate with an increased incidence of perioperative bleeding requiring massive transfusion, acute-on-chronic liver failure (ACLF), hepatorenal syndrome, sepsis caused by resistant organisms, and prolonged mechanical ventilation [7,10,12,13]. Furthermore, CTP classification influences optimal operative timing. CTP A cases generally tolerate elective scheduling, whereas CTP B cases warrant accelerated preoperative optimization or consideration of transcatheter alternatives, such as transcatheter aortic valve implantation (TAVI) or transcatheter edge-to-edge repair (TEER) [4,5]. CTP C represents a relative contraindication except in life-threatening scenarios requiring urgent intervention, where outcomes remain poor despite aggressive perioperative support [5,12,13].

However, these mortality estimates are derived from heterogeneous cohorts spanning two decades and may not fully reflect contemporary practice. Recent advances in perioperative management, including improved anesthetic techniques, goal-directed hemodynamic therapy, widespread adoption of viscoelastic-guided transfusion strategies, enhanced critical care protocols, and selective use of transcatheter alternatives, have likely improved outcomes, particularly in CTP A and B patients. Therefore, contemporary mortality rates in high-volume centers with dedicated multidisciplinary teams may be lower than these historical benchmarks. Nevertheless, large-scale validation studies are still needed to establish updated mortality estimates [14].

#### 2.1.3 MELD Score: Superior Risk Prediction for Cardiac Surgery

The MELD score demonstrates superior predictive accuracy compared with CTP for forecasting perioperative outcomes in cardiac surgery [4,5,15]. Patients with MELD scores <13 achieve perioperative mortality comparable to that of non-cirrhotic populations when appropriately optimized, with complications that are primarily cardiac rather than hepatic in nature [4,5]. Patients with MELD scores 13–18 face escalating mortality (15%–35%) with a high incidence of acute kidney injury, sepsis, hepatic decompensation, and coagulopathy-related bleeding complications requiring aggressive transfusion strategies [4,5,7,10,15]. MELD scores >18–20 represent a prohibitive risk, with mortality exceeding 50%–70%, driven by severe hepatorenal dysfunction, profound coagulopathy, hemodynamic instability refractory to vasopressor support, and rapid hepatic decompensation [5,6,7,10,15,16]. These mortality ranges are wide and derive from heterogeneous retrospective studies conducted over the past two decades. Advances in surgical techniques, including increased use of off-pump coronary artery bypass grafting (OPCAB), and transcatheter valve interventions, as well as anesthetic management, such as improved hemodynamic monitoring and optimized fluid strategies, and perioperative care, including early renal replacement therapy, enhanced infection control, and multidisciplinary team management, have likely reduced mortality in contemporary practice, particularly among patients with MELD scores ≤15. However, the extent of this improvement remains uncertain, as most recent studies continue to report elevated mortality in patients with MELD scores >15–18, suggesting that fundamental hepatic dysfunction continues to drive adverse outcomes despite modern advances [17,18].

Importantly, MELD-Na, which incorporates serum sodium concentration, further refines risk stratification; hyponatremia (<130 mEq/L) indicates severe portal hypertension and portends particularly poor prognosis, including increased mortality and multiorgan failure [10,11,16]. The objective nature of MELD variables, derived from routine laboratory parameters, enables rapid, reproducible risk assessment and facilitates meaningful prognostic discussions with patients and families regarding realistic surgical candidacy and alternative therapeutic options [9,11].

### 2.2 Advanced Predictive Models: VOCAL-Penn and Mayo Scores

#### 2.2.1 VOCAL-Penn Cirrhosis Surgical Risk Score

Traditional scoring systems, including CTP, MELD, and MELD-Na, were not specifically developed for surgical populations and lack surgery-specific variables. The VOCAL-Penn score was derived and validated using data from 3785 veterans with cirrhosis who underwent major surgery across 128 US medical centers, using multivariable logistic regression to predict 30-, 90-, and 180-day postoperative mortality [19].

The VOCAL-Penn model incorporates nine variables: age, American Society of Anesthesiologists (ASA) classification (2–4), surgery category, including chest/cardiac procedures, emergency surgery indication, non-alcoholic fatty liver disease (NAFLD) cirrhosis as the underlying etiology, albumin, platelet count, total bilirubin, and obesity (body mass index (BMI) ≥30). Notably, the inclusion of surgery-specific categories represents a major advance over traditional liver-only scores. In validation cohorts, VOCAL-Penn demonstrated superior discrimination compared with existing tools, with a C-statistic of 0.859 for 30-day mortality versus 0.766 for the Mayo score and 0.752 for MELD-Na (*p* = 0.003). Calibration was excellent across the entire risk spectrum, whereas the Mayo score tended to overestimate mortality. The VOCAL-Penn calculator is freely available online (https://www.vocalpennscore.com) and should be considered for preoperative risk stratification in cirrhotic patients undergoing cardiac surgery [19].

However, important limitations regarding generalizability must be acknowledged. The VOCAL-Penn derivation cohort consisted of 3785 predominantly male veterans (>90% male) from United States (US) Veterans Affairs medical centers, which may limit applicability to female patients, non-Western populations, and civilian healthcare systems with different practice patterns and patient demographics. The model was developed across a range of surgical procedures, including abdominal, orthopedic, and cardiothoracic operations, rather than exclusively for cardiac surgery, which may limit the precision of the model when applied to cardiac surgical cohorts specifically. External validation in diverse populations, including female patients, non-veteran cohorts, and international settings, is needed to confirm the generalizability of the model across demographic and geographic contexts [19].

#### 2.2.2 Mayo Risk Score

The Mayo risk score incorporates MELD score, age, ASA class, and cirrhosis etiology to predict postoperative mortality. In a large study of patients undergoing major cardiac and abdominal surgery, the Mayo score was the best predictor of 1-month and 3-month mortality, with mortality increasing alongside MELD score, age, and ASA class [20]. However, recent evidence suggests that the Mayo score may overestimate surgical risk in contemporary practice because advances in medical and surgical management have improved overall outcomes [21]. Nevertheless, the Mayo score remains a valuable tool for initial risk assessment when integrated with other clinical parameters.

These characterizations are not contradictory but reflect different eras and comparators: in its original 2007 derivation cohort, the Mayo score was the best available predictor of short-term mortality among the tools then in use, whereas more recent head-to-head comparisons in contemporary cohorts indicate that it tends to overestimate risk relative to newer, surgery-specific models such as VOCAL-Penn [19,21].

#### 2.2.3 Comparative Performance

In head-to-head comparisons, VOCAL-Penn demonstrates superior discriminative ability and calibration compared with Mayo, MELD, and MELD-Na scores across multiple postoperative time points [19]. The addition of surgery-specific variables, such as procedure type and emergency indication, as well as cirrhosis-specific factors, including NAFLD etiology and obesity, likely accounts for the improved performance of the metric. For cardiac surgery specifically, including chest and cardiac procedures as a distinct surgical category in VOCAL-Penn yields more accurate risk estimates than general surgical scores. However, it is important to note that VOCAL-Penn was derived from a predominantly male veteran population undergoing various major surgeries, not exclusively cardiac procedures, which may limit the generalizability of the model to specific cardiac surgical cohorts.

#### 2.2.4 External Validation of Risk Prediction Models in Contemporary Cardiac Surgery Cohorts

Recent external validation studies have begun to assess the performance of existing risk prediction models specifically in cirrhotic patients undergoing cardiac surgery, providing critical insights into model accuracy in contemporary practice. Jenkins et al. [22] conducted a single-center retrospective validation study evaluating the performance of the Mayo risk score and VOCAL-Penn score in 220 cirrhotic patients who underwent cardiac surgery. This study represents an important contemporary validation of risk prediction models specifically in the cardiac surgery population.

The key finding from Jenkins et al. [22] was that both the Mayo score and VOCAL-Penn substantially overestimated postoperative mortality in this cohort. The Mayo score predicted a mortality of 20.9%, and VOCAL-Penn predicted a mortality of 9.0%, whereas observed mortality was markedly lower, at 4.1% at 30 days and 12.6% at 90 days. This discrepancy suggests that, at least in some contemporary single-center cohorts, both established risk models may not be well calibrated and can meaningfully overstate perioperative risk.

These validation findings have important clinical implications. First, clinicians should be cautious when using Mayo or VOCAL-Penn predicted mortality as an absolute figure for patient counseling, since observed outcomes in this cohort were substantially better than predicted. Second, the overestimation observed by Jenkins et al. [22] reinforces the broader point made in Section 2.1.2 that historical mortality benchmarks may not reflect contemporary outcomes with modern perioperative care. Third, this single-center experience should not be over-generalized: larger, multicenter, and prospective validation studies remain necessary to determine whether this degree of overestimation is consistent across practice settings, procedure types, and cirrhosis severities. Finally, no cardiac-surgery-specific model has yet demonstrated consistently accurate calibration across independent cohorts, underscoring the need for further recalibration or development of surgery type-specific tools [17].

#### 2.2.5 Heart–Liver Team: Mandatory Multidisciplinary Discussion

Cardiac surgery in cirrhotic patients requires comprehensive, multidisciplinary evaluation and collaborative decision-making involving a cardiothoracic surgeon, interventional cardiologist, hepatologist, cardiac anesthesiologist, intensivist, and, when appropriate, transplant surgeon [5,16,19]. This structured team approach addresses critical questions that isolated specialists cannot adequately resolve independently [15,16].

**Fig. 1.**
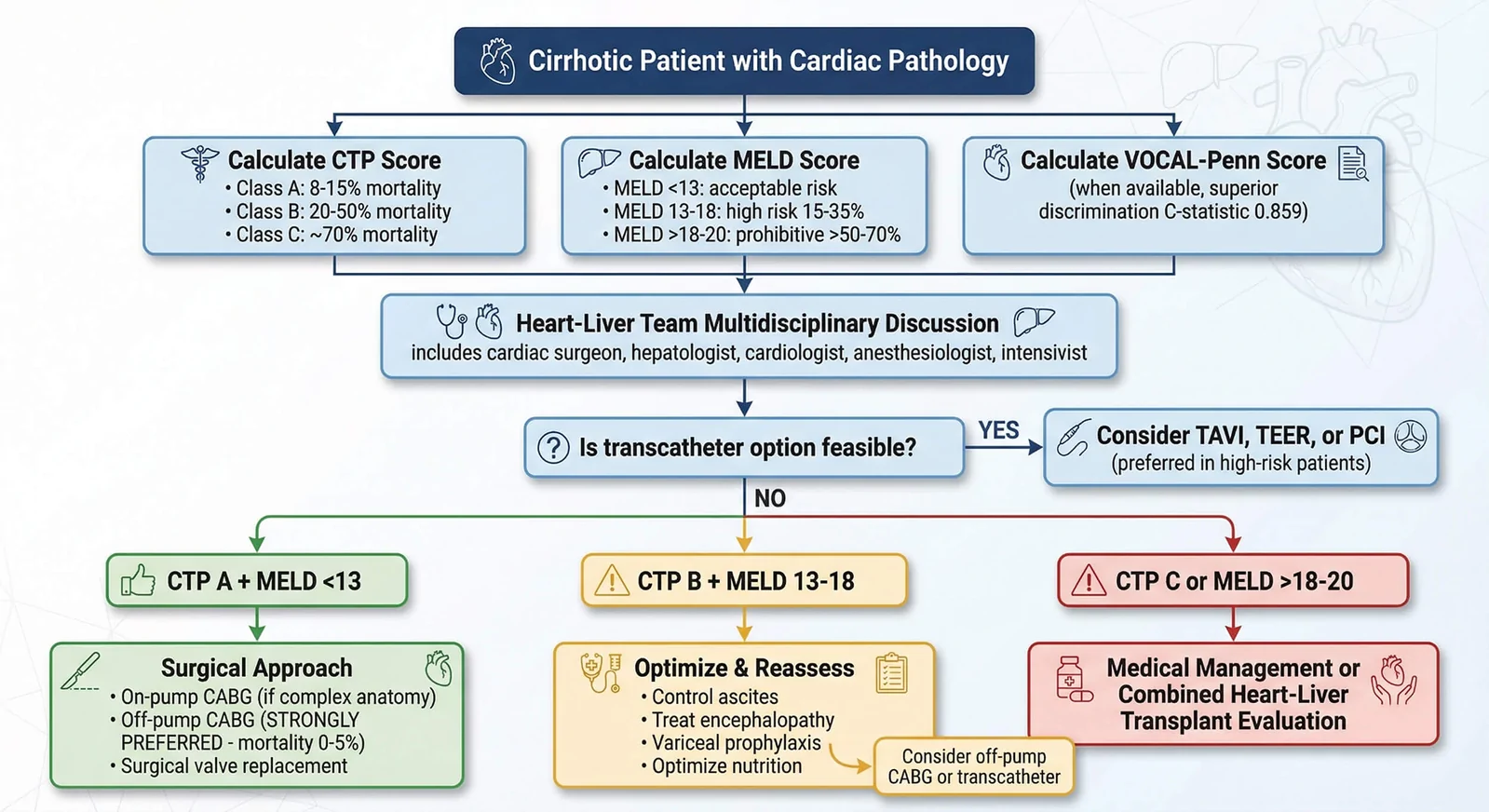
**Clinical decision-making algorithm for patients with concomitant cardiac and liver disease**. Assessment using CTP, MELD, and VOCAL-Penn scores is followed by multidisciplinary Heart–Liver Team evaluation to determine suitability for surgery, transcatheter intervention, medical optimization, conservative management, or liver transplant assessment. CTP, Child-Turcotte-Pugh; MELD, Model for End-Stage Liver Disease; VOCAL-Penn, Veterans Outcomes and Costs Associated with Liver Disease – Penn.

The Heart–Liver Team should systematically evaluate disease severity using both cardiac parameters (coronary anatomy, ventricular function) and hepatic parameters (CTP, MELD, and VOCAL-Penn or Mayo scores), determine the true urgency and clinical necessity of cardiac intervention, and carefully assess whether transcatheter alternatives (TAVI, TEER, percutaneous coronary intervention (PCI)) might achieve therapeutic goals while avoiding operative risk entirely [5,9,11,19]. The team should also evaluate candidacy for combined heart–liver transplantation as a potential definitive solution, particularly for younger cirrhotic patients with otherwise suitable hemodynamics [16,20]. Critically, the team should formulate realistic mortality and morbidity estimates, neither catastrophizing nor providing false reassurance, and ensure that patients and families understand both the substantial perioperative risks inherent to cardiac surgery in cirrhosis and the potential for meaningful long-term benefit in appropriately selected candidates [5,16]. This collaborative deliberation model helps prevent both unsafe overtreatment and inappropriate denial of potentially beneficial surgery in suitable patients [5,19]. The integrated decision-making pathway, from risk stratification through multidisciplinary discussion to selection of surgical, transcatheter, or medical management, is summarized in Fig. 1.

### 2.3 Preoperative Evaluation and Optimization

#### 2.3.1 Comprehensive Hepatic Assessment

A comprehensive clinical history and physical examination form the foundational elements of hepatic assessment in patients with suspected or confirmed cirrhosis [16]. The clinician should meticulously document the underlying etiology of hepatic dysfunction, determining whether cirrhosis is attributable to alcohol-associated liver disease, viral hepatitis (hepatitis B or C), autoimmune hepatitis, or metabolic etiologies, including NAFLD, hemochromatosis, Wilson disease, or primary biliary cholangitis [20]. Careful documentation of all prior episodes of hepatic decompensation is recommended, including variceal bleeding from esophageal or gastric varices, any history of hepatic encephalopathy regardless of grade, and the current or prior presence of ascites, as these historical features provide critical insight into disease severity and perioperative risk [16,20,21]. The physical examination should systematically assess for the presence of cirrhotic stigmata, including careful evaluation for jaundice indicating hyperbilirubinemia, spider angiomas reflecting portal hypertension and hyperdynamic circulation, palmar erythema suggesting hepatic dysfunction, and quantification of ascites severity through percussion and palpation techniques [16,21].

Laboratory evaluation is essential for objective assessment of hepatic synthetic function and should include measurement of serum albumin, total and direct bilirubin, prothrombin time and INR, platelet count, serum sodium concentration, serum creatinine, and serum prealbumin as a short-term nutritional marker [9,10,11,16]. Both the CTP score and the MELD/MELD-Na scores should be calculated and precisely documented in the medical record, as these complementary scoring systems provide independent risk stratification for perioperative outcomes [10,11]. Scores should be recalculated if a significant temporal interval elapses between the initial laboratory assessment and the scheduled surgical procedure, as dynamic changes in hepatic and renal function may substantially modify the perioperative risk profile [9,10,11]. When available, VOCAL-Penn or Mayo scores should also be calculated to provide surgery-specific risk estimates.

Radiological imaging, utilizing either abdominal ultrasonography with Doppler flow evaluation or contrast-enhanced computed tomography (CT), should be obtained to assess hepatic parenchymal morphology, including evaluation for cirrhotic features such as nodular contours, heterogeneous echogenicity, and architectural distortion [16]. The portal vein should be carefully evaluated on Doppler interrogation for caliber, luminal diameter, direction, and velocity of portal venous flow, and for the presence of acute or chronic portal vein thrombosis, which would significantly impact operative feasibility [23]. Additional imaging findings should include splenic size as a marker of portal hypertension-related splenomegaly, volume of ascites, and systematic surveillance for hepatocellular carcinoma or other hepatic malignancies, as the detection of neoplastic disease may substantially modify surgical planning and prognosis [16,24].

Esophagogastroduodenoscopy should be performed as a screening procedure to identify the presence and grade the severity of esophageal varices, as variceal hemorrhage represents one of the most catastrophic perioperative complications in cirrhotic patients [24]. In patients with large esophageal varices, identified during endoscopic examination, prophylactic intervention is strongly recommended before elective surgical procedures to substantially reduce the risk of massive variceal bleeding. Prophylactic interventions may include either endoscopic variceal band ligation or initiation of non-selective beta-adrenergic antagonist therapy, such as propranolol or carvedilol, to reduce portal venous pressure gradients [25].

#### 2.3.2 Cardiac Evaluation With Cirrhotic Cardiomyopathy Screening

Standard cardiac evaluation, including a 12-lead electrocardiogram (ECG), coronary angiography, and transthoracic echocardiography, forms the foundation of cardiac assessment in cirrhotic patients being considered for surgery [6,16]. These diagnostic modalities should provide detailed quantification of left ventricular systolic and diastolic function, comprehensive characterization of right ventricular performance, assessment of diastolic parameters reflective of left ventricular filling characteristics, and measurement of pulmonary artery pressures as indicators of right ventricular afterload and pulmonary hemodynamics [16,25,26]. Particular clinical attention should be directed toward the identification and characterization of cirrhotic cardiomyopathy, a distinct pathophysiological entity characterized by myocardial dysfunction in advanced hepatic disease, with profound implications for perioperative risk stratification and adverse cardiac outcomes [6,25].

Diastolic dysfunction represents a cardinal feature of cirrhotic cardiomyopathy and should be comprehensively assessed [25,26]. The tissue Doppler imaging-derived ratio of early mitral inflow velocity to early diastolic mitral annular velocity (E/e' ratio) is a sensitive and specific marker of diastolic dysfunction, with values exceeding 14 indicating abnormal diastolic properties and substantially elevated left ventricular filling pressures with important prognostic implications [26]. Additional parameters for assessment of diastolic dysfunction include quantification of the left atrial volume index normalized to body surface area, detailed characterization of mitral inflow characteristics such as the E/A ratio and deceleration time, and measurement of left atrial longitudinal strain using speckle-tracking echocardiography to detect subtle atrial dysfunction and predict perioperative decompensation [25,26].

Right ventricular systolic dysfunction is common in advanced cirrhosis and is characterized by impaired contractility, elevated afterload secondary to pulmonary hypertension, and limited tolerance of the hemodynamic stresses of surgery and anesthesia [6,25]. Comprehensive evaluation of right ventricular systolic function should incorporate measurement of tricuspid annular plane systolic excursion (TAPSE), tissue Doppler-derived tricuspid annular systolic velocity (S') as an independent marker of right ventricular contractility, and estimation of right ventricular systolic pressure derived from the peak tricuspid regurgitation jet velocity [27]. Assessment of the inferior vena cava (IVC) provides critical information regarding right atrial pressure and right ventricular function; an IVC diameter greater than 2.1 cm with less than 50% inspiratory collapse suggests markedly elevated right atrial pressure, severe right ventricular dysfunction, and poor prognosis [27].

Cardiac biomarkers, specifically B-type natriuretic peptide (BNP) and N-terminal pro-BNP (NT-proBNP), are useful markers of myocardial dysfunction and independent predictors of perioperative complications in cirrhotic patients [25]. Elevated preoperative concentrations of BNP and NT-proBNP correlate strongly with the severity of cirrhotic cardiomyopathy as manifested by diastolic dysfunction and elevated ventricular filling pressures, and independently predict the occurrence of major perioperative cardiac complications [25]. Dobutamine stress echocardiography, when technically feasible and clinically indicated, represents a valuable adjunctive diagnostic modality for further risk stratification in cirrhotic patients with suspected cardiomyopathy [6,25]. A blunted inotropic response to increasing doses of dobutamine suggests severe underlying cardiomyopathy and substantially impaired myocardial reserve. Such findings raise significant clinical concern regarding the ability of the patient to tolerate surgical hemodynamic stress and may warrant postponement or cancellation of elective procedures [25].

#### 2.3.3 Ascites and Portal Hypertension Management

Ascites should be controlled preoperatively through dietary sodium restriction and diuretic therapy, typically spironolactone with or without furosemide. Large-volume paracentesis with albumin replacement may be required for tense ascites. Uncontrolled ascites increases intra-abdominal pressure, impairs diaphragmatic excursion and ventilation, and predicts higher rates of postoperative respiratory complications and prolonged mechanical ventilation. Clinicians should exercise careful clinical judgment to avoid excessive diuresis, as overly aggressive fluid removal may precipitate hepatorenal syndrome [10].

Non-selective beta-adrenergic blockade is the cornerstone of pharmacological management of portal hypertension and should be achieved with agents such as carvedilol or propranolol to reduce resting heart rate. Beta-blockers reduce portal venous pressure gradients and substantially reduce the risk of perioperative variceal hemorrhage; however, beta-blockade may complicate intraoperative hemodynamic management and blunt compensatory tachycardic responses to hypovolemia or reduced cardiac output. Prophylactic variceal band ligation should be considered as an alternative or complementary approach in patients with large esophageal varices who cannot tolerate or do not adequately respond to beta-blocker therapy [21].

#### 2.3.4 Coagulation and Hematologic Optimization

A critical principle in the management of coagulation abnormalities in cirrhotic patients is that mild-to-moderate INR elevation in the range of 1.5 to 1.8 should not be routinely corrected with empiric fresh frozen plasma (FFP) administration, as this range reflects a rebalanced hemostatic state characteristic of advanced cirrhosis [7]. Routine correction of mildly elevated INR with FFP transfusions has been shown to worsen patient outcomes through multiple mechanisms, including iatrogenic volume overload, exacerbation of ascites formation, and increased risk of hepatic decompensation [28].

Vitamin K supplementation may be administered intravenously when there is clinical suspicion of cholestasis or vitamin K deficiency. However, vitamin K-refractory coagulopathy reflects impaired synthetic function rather than nutritional deficiency. Platelet transfusion should be considered in cirrhotic patients presenting with platelet counts below 50,000 cells per μL when active bleeding is present or anticipated. However, routine prophylactic platelet transfusion in asymptomatic patients with moderately reduced platelet counts is not indicated [7,28].

Viscoelastic testing modalities, specifically thromboelastography (TEG) or rotational thromboelastometry (ROTEM), represent superior diagnostic techniques compared to conventional laboratory coagulation testing when available. These point-of-care tests characterize the specific nature of coagulation defects by assessing the kinetics of clot formation, clot strength, and clot dissolution in whole blood samples, thereby enabling more individualized and targeted transfusion strategies. Utilization of viscoelastic testing to guide transfusion therapy has been demonstrated to reduce unnecessary transfusions, minimize iatrogenic volume overload, prevent exacerbation of ascites, and improve overall perioperative outcomes [28,29].

#### 2.3.5 Hepatic Encephalopathy, Infection, and Nutrition Optimization

Hepatic encephalopathy should be managed with lactulose and rifaximin as needed to achieve clinical improvement. Preoperative encephalopathy indicates advanced hepatic dysfunction, predicts difficult weaning from mechanical ventilation, and is associated with increased postoperative delirium and ICU complications. Identification and correction of precipitating factors for hepatic encephalopathy are essential and should include evaluation for constipation, occult infection, intravascular volume depletion, and acute renal failure [16,30]. Sedating medications should be carefully avoided in cirrhotic patients with encephalopathy [16].

Comprehensive infection screening should be performed preoperatively in all cirrhotic patients, including urinalysis and urine culture, blood cultures, chest radiography, and ascitic fluid analysis when large-volume ascites is present. This systematic screening approach facilitates early detection of occult infections, which may be clinically silent in cirrhotic patients due to impaired immune function and reduced inflammatory responses [16].

High-protein dietary support should be implemented to preserve skeletal muscle mass and maintain hepatic protein synthesis, as protein malnutrition is prevalent in cirrhotic patients and significantly worsens perioperative outcomes. Energy-dense nutritional supplementation should be administered to optimize metabolic substrate availability and support hepatic regenerative capacity. Prehabilitation programs incorporating ambulation and respiratory exercises should be initiated when time permits, before surgical intervention, as physical conditioning improves functional capacity and may reduce perioperative morbidity and mortality. Serial monitoring of serum prealbumin concentration should be performed to assess the adequacy of nutritional support [31].

### 2.4 Intraoperative Management

#### 2.4.1 Anaesthetic Strategy

Selection of anesthetic agents in cirrhotic patients should prioritize the use of short-acting drugs with hepatic-sparing properties to minimize accumulation and toxicity in patients with severely compromised hepatic metabolism [16,32,33]. For induction of general anesthesia, remifentanil is an excellent choice because the drug undergoes rapid ester metabolism by plasma esterases, independent of hepatic function [32,34]. Low-dose propofol may also be used for induction, although dosing should be substantially reduced from standard recommendations because cirrhotic patients often demonstrate exaggerated hemodynamic responses [25,32,33]. Alternatively, ketamine may be useful in hemodynamically compromised cirrhotic patients due to the associated sympathomimetic properties [32,33].

Maintenance of general anesthesia should be achieved with a combination of volatile anesthetic agents, such as sevoflurane or isoflurane, which undergo minimal hepatic metabolism, together with a continuous remifentanil infusion [32,33]. Neuromuscular blockade should preferentially be achieved through administration of cisatracurium or atracurium, which undergo Hofmann elimination and ester hydrolysis that are independent of hepatic and renal organ function [32,33]. Rocuronium and vecuronium should generally be avoided in cirrhotic patients, as these agents accumulate to toxic levels and cause prolonged neuromuscular blockade [32,33].

#### 2.4.2 Hemodynamic Monitoring and Goals

Comprehensive hemodynamic monitoring should be established in all cirrhotic patients undergoing surgery and should include placement of an arterial catheter for beat-to-beat blood pressure monitoring, insertion of a central venous catheter for central venous pressure measurement, and consideration of a pulmonary artery catheter in selected high-risk cases [32,33,35]. Intraoperative transoesophageal echocardiography (TEE) should be employed when feasible to directly assess ventricular function and detect regional wall motion abnormalities [32,33,36].

Specific hemodynamic targets should be maintained throughout the intraoperative period to optimize tissue perfusion and prevent end-organ injury. Mean arterial pressure (MAP) should be maintained at 60–70 mmHg, as hypotension below this range may cause severe hepatic and renal hypoperfusion [10,35]. Central venous pressure should be maintained at 6–10 mmHg, as levels above 12 mmHg may cause passive hepatic congestion [37]. Cardiac index should remain above 2.0 L/min/m^2^, urine output should exceed 0.5 mL/kg/h [10,37], and core body temperature should be maintained between 35.5 °C and 37 °C throughout the operative procedure [32,33].

#### 2.4.3 Vasopressors and Inotropes

Norepinephrine serves as the first-line vasopressor agent in hemodynamically compromised cirrhotic patients and should be administered to counteract the systemic vasodilation and reduced systemic vascular resistance characteristic of advanced cirrhosis [6,16,25,35,38]. Inotropic support should be provided by administering dobutamine or milrinone in patients with low cardiac output [6,25,32,33,35]. Combined vasopressor and inotropic therapy is often required in critically ill cirrhotic patients [6,16,25,32,33,35,38].

#### 2.4.4 Cardiopulmonary Bypass Strategy: Minimize Duration and Injury

The total duration of CPB should be minimized and kept below 100 to 120 minutes, as CPB times longer than 150 minutes are associated with substantially increased perioperative complications [8,12]. Aortic cross-clamping time should be maintained below 90 minutes when surgically feasible [39]. Off-pump coronary artery bypass grafting (OPCAB) is strongly preferred in cirrhotic patients whenever cardiac anatomy permits, as off-pump approaches eliminate CPB and are associated with substantially lower mortality rates [5,40].

#### 2.4.5 Why Off-Pump CABG is Superior in Cirrhosis

Observational cohort data demonstrate a dramatic reduction in mortality with off-pump versus on-pump CABG in cirrhotic populations: off-pump approaches are associated with mortality rates of 0%–5% in CTP A/B patients, compared with 10%–30% for conventional on-pump CABG in similar cohorts [5,40]. The mechanisms underlying this apparent benefit are multifactorial: (1) avoidance of CPB-induced systemic inflammatory response syndrome (SIRS), which is markedly exaggerated in cirrhosis; (2) elimination of hemodilution-related coagulopathy and reduced transfusion requirements; (3) avoidance of CPB-related hepatic ischemia-reperfusion injury; (4) preservation of endogenous coagulation factors with less heparin/protamine exposure; (5) reduced renal injury from CPB-associated inflammatory mediators and hypoperfusion [5,40]. However, these findings are derived from retrospective series with inherent selection bias; patients selected for off-pump approaches may have had less complex coronary anatomy, better hepatic reserve, or other favorable characteristics. No randomized controlled trials exist, and questions remain regarding the optimal completeness of revascularization and applicability across the full spectrum of cirrhosis severity (see Controversies section).

Minimally invasive surgical approaches, such as TAVI and TEER, avoid the need for CPB and should be prioritized in high-risk cirrhotic patients when anatomically feasible [19].

#### 2.4.6 Cardiopulmonary Bypass Parameters

CPB pump flows should be maintained at a minimum of 2.3 L/min/m^2^ throughout the bypass period to ensure adequate perfusion of the hepatic and renal circulations [39]. MAP during CPB should be maintained at or above 50–60 mmHg, and prolonged periods of hypotension during bypass should be avoided [35,39]. Hematocrit should be maintained at a minimum level of 24%–25% during CPB to prevent tissue hypoxia [39]. Moderate hypothermia with core body temperatures in the range of 32 °C to 34 °C is acceptable during CPB, although profound hypothermia should be avoided [39]. Albumin-containing priming solutions may be considered instead of exclusively crystalloid-based solutions to reduce hemodilution and help preserve colloid osmotic pressure [41].

#### 2.4.7 Intraoperative Bleeding and Transfusion: Viscoelastic-Guided Strategy

Antifibrinolytic therapy using tranexamic acid (TXA) should be administered intraoperatively in cirrhotic patients to reduce fibrinolysis and minimize perioperative hemorrhage [7,42]. Multiple clinical trials have shown that TXA substantially reduces intraoperative bleeding and the need for perioperative blood transfusion [42]. Intraoperative cell salvage technology should be employed whenever available and feasible, thereby substantially reducing the need for transfusion of banked allogeneic blood products [43].

Viscoelastic testing modalities, specifically TEG or ROTEM, provide superior guidance for targeted hemostatic therapy than conventional laboratory coagulation tests and should be used to guide transfusion decisions throughout the intraoperative period [28,44]. When viscoelastic testing demonstrates prolonged R time, indicating delayed initiation of clot formation, FFP or prothrombin complex concentrate should be administered [29,44]. When testing demonstrates prolonged K time or a low angle, indicating impaired clot formation rate and reduced fibrinogen availability, fibrinogen supplementation should be provided through cryoprecipitate or fibrinogen concentrate [28,44]. When testing demonstrates low maximum amplitude, indicating impaired clot strength, platelet transfusion or desmopressin should be considered [28,29,44]. When testing demonstrates elevated clot lysis at 30 minutes (LY30) indicating excessive fibrinolysis, antifibrinolytic therapy should be administered [7,42,44].

Red blood cell transfusion should be guided by a restrictive transfusion strategy with hemoglobin targets of 8–9 g/dL [28,45]. When major bleeding is anticipated, a massive transfusion protocol using balanced ratios of red blood cells, FFP, and platelets should be initiated proactively [44].

Heparin reversal after CPB should be achieved with careful, titrated administration of protamine sulfate [8,44]. Repeat viscoelastic testing after protamine should be performed to assess the adequacy of heparin reversal and guide any additional hemostatic support [29,44].

### 2.5 Postoperative Management

#### 2.5.1 Intensive Care Unit Monitoring and Early Detection of Hepatic Decompensation

All cirrhotic patients undergoing cardiac surgical procedures should be admitted to an ICU with specialized expertise in the management of combined cardiac and hepatic failure [16,35,38]. Continuous hemodynamic and physiological monitoring should be maintained throughout the postoperative period, with hourly urine output documentation and daily laboratory assessments, including a complete blood count, a comprehensive metabolic panel, the INR, and lactate [10,16,35,38]. Serial monitoring of these parameters enables early detection of deterioration and allows for timely therapeutic interventions [16,38].

Progressive elevation of serum bilirubin concentration should be closely monitored, as rising bilirubin levels indicate severe hepatocellular necrosis and impending liver failure [13,16]. Acute INR elevation in the absence of concurrent active bleeding is a critical warning sign of deteriorating hepatic synthetic function [16]. Worsening hepatic encephalopathy indicates accumulation of neurotoxic metabolites and represents a marker of severe hepatic dysfunction [13,30]. Escalating vasopressor requirements despite adequate intravascular volume status indicate progressive vasodilatory shock [13,38]. Oliguria, defined as urine output below 0.5 mL/kg/h despite optimization, indicates acute kidney injury and suggests the development of hepatorenal syndrome [10,13].

The combination of findings, including hyperbilirubinemia, elevated INR, and acute kidney injury in a postoperative cirrhotic patient, constitutes ACLF and represents a life-threatening condition [13]. Recognition of this syndrome should prompt urgent consultation with hepatology and liver transplantation specialists [38].

#### 2.5.2 Hemodynamic and Fluid Management

Postoperative hemodynamic targets should include a MAP of 60–70 mmHg, central venous pressure of 6–10 mmHg, urine output greater than 0.5 mL/kg/h, and cardiac index above 2.0 L/min/m^2^ [46,47,48].

A restrictive fluid strategy should be employed, minimizing administration of crystalloid solutions and utilizing vasopressors rather than repeated fluid boluses to maintain adequate MAP [47,48,49]. Once hemodynamic stability is achieved, central venous pressure should be maintained at low-normal levels to prevent reaccumulation of ascites [46,49]. Diuretic therapy with furosemide, with or without spironolactone, should then be initiated to achieve negative fluid balance [46,49]. Nephrotoxic agents, including nonsteroidal anti-inflammatory drugs, aminoglycosides, and iodinated contrast, should be scrupulously avoided [10,46].

#### 2.5.3 Acute Kidney Injury Prevention

Acute kidney injury occurs in up to 80% of cirrhotic patients undergoing cardiac surgical procedures and represents a powerful independent predictor of poor postoperative outcomes [5,10,12,16]. Adequate renal perfusion pressure should be maintained by targeting a minimum MAP of 60–70 mmHg [35,37,38,46]. Cardiac output should be optimized with inotropic agents when necessary [35,38]. Positive fluid balance should be minimized by favoring vasopressor support over repeated fluid boluses [35,37,38,46]. Central venous pressure should be kept at low-normal levels of 6–10 mmHg [35,37,38]. Nephrotoxic medications should be scrupulously avoided [10,16,46]. Early initiation of continuous venovenous hemofiltration (CVVH) should be considered once clinical and biochemical evidence of acute kidney injury appears, rather than delaying renal replacement therapy until severe azotemia or hyperkalemia develops [10,50,51,52]. Early renal replacement therapy has been associated with improved perioperative outcomes and reduced mortality [50,51,52].

#### 2.5.4 Infection Prevention and Early Treatment

Comprehensive infection prevention bundles should be implemented for all cirrhotic cardiac surgery patients, including meticulous hand hygiene protocols, systematic central venous line care, and ventilator care bundles [16,53,54]. Urinary catheters and central venous lines should be removed as soon as clinically feasible [53,54]. Antibiotic stewardship principles should be applied, with prophylactic antibiotics typically discontinued after 24 hours in the absence of established infection [55].

A low threshold for empiric antibiotic initiation should be maintained in cirrhotic patients with suspected infection, as immune dysfunction and reduced inflammatory responses may permit rapid progression to sepsis [16]. Typical empiric regimens pending culture results include ceftriaxone or cefepime combined with a fluoroquinolone [16,55]. Sepsis occurs in 30%–50% of cirrhotic patients undergoing cardiac surgical procedures and represents one of the leading causes of perioperative mortality [5,12].

#### 2.5.5 Thromboprophylaxis and Anticoagulation Balance

Venous thromboembolism (VTE) prophylaxis with unfractionated heparin or mechanical prophylaxis, such as sequential compression devices, should be initiated once hemostasis is secured [28,56]. For anticoagulation in patients with a prosthetic valve or atrial fibrillation, warfarin is preferred over direct oral anticoagulants (DOACs) in cirrhotic patients; lower INR targets may be considered with careful monitoring [19,28,57]. Bioprosthetic valves are strongly preferred in cirrhotic patients to avoid the burden of long-term anticoagulation, which is particularly challenging given the rebalanced hemostatic state and unpredictable INR responses in cirrhosis [19].

#### 2.5.6 Early Mobilization, Nutrition, and Discharge Planning

Respiratory physiotherapy and spirometry measurements should be initiated on postoperative day 1 [58,59]. Patients should be sitting at the bedside on postoperative day 1, with progression to standing and ambulation by postoperative days 2 to 3 if hemodynamically stable [58,59]. Early extubation should be targeted within 24 to 48 hours after surgery when feasible [53,59].

Enteral nutrition is strongly preferred over total parenteral nutrition and should be delivered via a nasogastric feeding tube or, if oral intake is inadequate, a post-pyloric feeding tube [16,31,60]. Nutritional goals should include provision of adequate protein and total energy intake to support hepatic regeneration and muscle protein synthesis [31,60]. Serial serum prealbumin measurements should be performed to assess the adequacy of nutritional support [16,31].

Comprehensive discharge planning should be established, with clear follow-up appointments, including a cardiothoracic surgery evaluation at 2 to 4 weeks postoperatively, a hepatology consultation at 1 to 2 weeks postoperatively, and ongoing cardiology follow-up [16,20]. Patients demonstrating persistent hepatic dysfunction despite appropriate perioperative management should be evaluated for potential liver transplantation [16,20].

Table 1 summarizes management.

**Table 1.** **Phase-oriented perioperative management algorithm**.

Phase	Category	Key actions and recommendations
Preoperative	Risk assessment	• Calculate CTP and MELD/MELD-Na; classify operability (CTP A vs. B vs. C). • Calculate the VOCAL-Penn score, when available: 9 variables including age, ASA (2–4), surgery category (chest/cardiac), emergency indication, NAFLD etiology, albumin, platelets, bilirubin, and obesity (BMI ≥30); superior discrimination (C-statistic 0.859) vs. Mayo (0.766) and MELD-Na (0.752). • Calculate the Mayo score: MELD + age + ASA class + cirrhosis etiology (may overestimate risk in contemporary practice). • Multidisciplinary Heart–Liver Team discussion: assess alternatives (TAVI, TEER, medical management, transplant). • Full hepatic evaluation: history, examination, labs (albumin, bilirubin, INR, platelets, Na, Cr). • Imaging: ultrasound/CT of the liver to assess the portal vein, ascites, and HCC. • Endoscopy for variceal grading: band ligation or beta-blocker prophylaxis if indicated.
Preoperative	Optimization	• Ascites: sodium restriction; diuretics (spironolactone ± furosemide); large-volume paracentesis + albumin. • Portal hypertension: non-selective beta-blockers (carvedilol or propranolol) for variceal prophylaxis. • Coagulation: avoid routine INR correction (1.5–1.8 acceptable); administer vitamin K if deficient; correct severe thrombocytopenia (<50,000/μL); viscoelastic testing (TEG/ROTEM) preferred. • Encephalopathy: lactulose + rifaximin; avoid sedatives • Infection: screen for and treat infection (blood/urine cultures; ascitic fluid analysis if needed). • Nutrition: high-protein diet (1.2–1.5 g/kg); prehabilitation; monitor prealbumin.
Intraoperative	Anesthesia	• Short-acting, hepatically-sparing agents: remifentanil, cisatracurium, atracurium. • Avoid hepatically metabolized drugs: morphine, rocuronium, volatile anesthetics (with prolonged use). • Invasive monitoring: arterial line, CVP, TEE; consider a PA catheter in high-risk cases.
Intraoperative	Hemodynamics and CPB	• Goals: MAP 60–70 mmHg, CVP 6–10 mmHg, CI >2.0 L/min/m^2^, UO >0.5 mL/kg/h, core temperature 35.5–37 °C. • Vasopressors: First-line norepinephrine (0.05–0.2 μg/kg/min); add dobutamine or milrinone for low output. • CPB strategy: Minimize duration (<100–120 min); off-pump CABG strongly preferred (mortality 0–5% vs. 10–30% with on-pump in CTP A/B). • Maintain flows ≥2.3 L/min/m^2^; target MAP 50–60 mmHg on pump; avoid profound hemodilution (Hct ≥24–25%). • Consider TAVI or TEER to avoid CPB entirely.
Intraoperative	Bleeding and transfusion	• Antifibrinolytics: Tranexamic acid (10–15 mg/kg bolus), followed by 1–2 mg/kg/h infusion. • Cell salvage: Use intraoperative cell salvage when available. • Viscoelastic-guided transfusion: TEG/ROTEM is superior to conventional tests for real-time guidance. • Restrictive RBC strategy: Maintain Hb at 8–9 g/dL; initiate a massive transfusion protocol if needed (1:1:1 or 2:1:1). • Careful protamine reversal: 0.5–1 mg/mL IV over 5–10 minutes.
Postoperative	ICU monitoring	• Admit to an ICU experienced in managing cardiac and hepatic failure. • Monitor urine output hourly; obtain daily labs (CBC, CMP, INR, lactate, troponin, bilirubin). • Red flags: rising bilirubin/INR, worsening encephalopathy, escalating vasopressor requirements, oliguria, elevated lactate. • ACLF (bilirubin ↑ + INR ↑ + AKI): obtain an urgent hepatology/transplant consult.
Postoperative	Hemodynamics and AKI	• Goals: MAP 60–70 mmHg, CVP 6–10 mmHg, CI >2.0 L/min/m^2^, UO >0.5 mL/kg/h. • AKI prevention: maintain renal perfusion, minimize positive fluid balance, and avoid nephrotoxins (NSAIDs, aminoglycosides, contrast). • Restrictive fluid strategy: use vasopressors rather than fluid boluses; target a low-normal CVP once stable. • Early RRT: initiate CVVH when AKI is evident rather than waiting for severe azotemia.
Postoperative	Infection and VTE	• Prevention bundles: hand hygiene, line care, ventilator bundle, and early catheter removal. • VTE prophylaxis: UFH 5000 units SC q8h once hemostasis is secured; mechanical prophylaxis (SCDs). • Low threshold for empiric antibiotics: ceftriaxone + fluoroquinolone or cefepime + fluoroquinolone pending cultures. • Anticoagulation balance: warfarin preferred (lower INR target: 2–2.5 for aortic valves, 2.5–3.5 for mitral valves); bioprosthesis preferred in cirrhotic patients.
Postoperative	Recovery and follow-up	• Mobilization: respiratory physiotherapy on day 1; standing and walking on days 2–3; target early extubation within 24–48 h. • Nutrition: enteral feeding preferred (1.2–1.5 g/kg protein, 25–35 kcal/kg); monitor prealbumin. • Discharge: cardiothoracic surgery (follow-up in 2–4 weeks), hepatology (in 1–2 weeks), cardiology (ongoing); consider transplant evaluation if liver dysfunction persists.

Abbreviations: ACLF, acute-on-chronic liver failure; AKI, acute kidney injury; ASA, American Society of Anesthesiologists; BMI, body mass index; CABG, coronary artery bypass grafting; CBC, complete blood count; CI, cardiac index; CMP, comprehensive metabolic panel; CPB, cardiopulmonary bypass; Cr, creatinine; CT, computed tomography; CTP, Child–Turcotte–Pugh; CVP, central venous pressure; CVVH, continuous venovenous hemofiltration; Hb, hemoglobin; HCC, hepatocellular carcinoma; Hct, hematocrit; ICU, intensive care unit; INR, international normalized ratio; IV, intravenous; MAP, mean arterial pressure; MELD, Model for End-Stage Liver Disease; MELD-Na, MELD-sodium; NAFLD, non-alcoholic fatty liver disease; NSAIDs, nonsteroidal anti-inflammatory drugs; PA, pulmonary artery; q8h, every 8 hours; RBC, red blood cell; ROTEM, rotational thromboelastometry; RRT, renal replacement therapy; SC, subcutaneous; SCD, sequential compression device; TAVI, transcatheter aortic valve implantation; TEE, transesophageal echocardiography; TEER, transcatheter edge-to-edge repair; TEG, thromboelastography; UFH, unfractionated heparin; UO, urine output; VTE, venous thromboembolism.

#### 2.5.7 Controversies and Future Directions

Despite growing clinical experience with cardiac surgery in cirrhotic patients, multiple areas of controversy and uncertainty persist, reflecting the limited high-quality evidence available in this population. Table 2 provides a structured summary of these major controversies, outlining the current evidence, specific knowledge gaps, and proposed study designs needed to address each unresolved clinical question.

**Table 2.** **Summary of major controversies and future research needs in cardiac surgery for cirrhotic patients**.

Controversy	Current evidence	Knowledge gaps	Proposed study design
Off-pump vs. on-pump CABG	Retrospective cohort studies suggest a mortality benefit with off-pump CABG (0–5% vs. 10–30% in CTP A/B patients). Proposed mechanisms include reduced systemic inflammation, preserved coagulation, and avoidance of CPB-related hepatic injury.	Selection bias limits interpretation; no RCTs exist; the optimal completeness of revascularization is unknown; applicability across all categories of cirrhosis severity remains unclear.	Multicenter RCT: Cirrhotic patients (MELD ≤15, CTP A/B) with suitable coronary anatomy were randomized to off-pump vs. on-pump CABG; primary outcome: 90-day mortality; secondary outcomes: hepatic decompensation, AKI, transfusion requirements, and graft patency at 1 year.
Optimal MELD threshold for surgical candidacy	Literature reports conflicting cutoffs, with MELD scores of 15–20 described as indicating “prohibitive risk”; this heterogeneity reflects differences in procedure type, era, and institutional experience.	No consensus threshold exists; studies lack standardized definitions of “acceptable risk”; transcatheter alternatives are not systematically considered.	International registry study: A prospective multicenter registry capturing all cirrhotic patients evaluated for cardiac surgery; tracking treatment decisions (surgery vs. transcatheter intervention vs. medical management) and outcomes stratified by MELD score; identifying the MELD threshold at which mortality exceeds benefit.
Timing of portal decompression (TIPS) before cardiac surgery	Potential benefits of preoperative TIPS include reduced portal hypertension, varices, and ascites; however, concerns remain regarding hepatic encephalopathy, cardiac overload, and unclear timing.	No studies have evaluated preoperative TIPS before cardiac surgery; the optimal timing is unknown; patient selection criteria remain undefined.	Pilot feasibility study: A prospective single-arm study of preoperative TIPS (performed 4–6 weeks before surgery) in CTP B patients with clinically significant portal hypertension undergoing elective cardiac surgery; assessing safety, portal pressure reduction, and perioperative outcomes.
Transcatheter vs. surgical valve interventions	TAVR and TEER avoid CPB and reduce perioperative mortality in high-risk patients; surgical AVR/MVR offers superior hemodynamic results and durability; however, comparative data in patients with cirrhosis are lacking.	No head-to-head comparisons exist; long-term durability of transcatheter interventions in cirrhosis is unknown; decision algorithms have not been validated.	Comparative effectiveness study: A multicenter prospective cohort comparing transcatheter (TAVR, TEER) vs. surgical valve interventions in patients with cirrhosis; matched on MELD, CTP, age, and valve pathology; outcomes: 30-day mortality, 1-year survival, valve hemodynamics, and reintervention rates.
Anticoagulation management	Cirrhotic patients exhibit “rebalanced hemostasis” with simultaneous risks of bleeding and thrombosis; conventional INR-based strategies may be inappropriate; viscoelastic testing provides a superior assessment.	Optimal anticoagulation strategies for mechanical valves and AF in cirrhosis remain undefined; bridging protocols are unclear; prediction of bleeding vs. thrombosis risk is imprecise.	Prospective cohort study with a biomarker substudy: Cirrhotic patients requiring postoperative anticoagulation; use viscoelastic testing (TEG/ROTEM) plus thrombin generation assays to guide anticoagulation; track bleeding and thrombotic events; develop cirrhosis-specific anticoagulation protocols.
Machine learning and cardiac surgery-specific risk models	Existing models (MELD, CTP, VOCAL-Penn, and Mayo) demonstrate variable performance; no cardiac surgery-specific ML model exists for cirrhotic patients; ML could integrate imaging, biomarkers, and functional testing.	Current models are based on traditional regression; lack integration of advanced variables, such as frailty, cardiac biomarkers, and hepatic imaging; validation in cardiac surgery populations remains incomplete.	ML model development study: multicenter cohort (>1000 patients) with comprehensive preoperative data (demographics, laboratory, imaging, frailty indices, and cardiac biomarkers); develop ML model for mortality and decompensation; perform external validation in independent cohorts; compare performance with VOCAL-Penn.
Frailty assessment	Frailty (LFI, FFI, CFS) predicts mortality in cirrhotic populations independently of MELD; captures functional reserve not reflected in biochemical scores; cardiac surgery-specific data are lacking.	Which frailty assessment tool is optimal for cirrhotic patients undergoing cardiac surgery? Does frailty improve risk stratification beyond VOCAL-Penn/MELD-Na? Can prehabilitation reduce frailty-related risk?	Prospective validation study: assess multiple frailty indices (LFI, FFI, CFS) in cirrhotic patients undergoing cardiac surgery; determine the incremental predictive value beyond existing scores; pilot a prehabilitation RCT in frail patients.

Abbreviations: AKI, acute kidney injury; AF, atrial fibrillation; AVR, aortic valve replacement; CABG, coronary artery bypass grafting; CFS, Clinical Frailty Scale; CPB, cardiopulmonary bypass; CTP, Child–Turcotte–Pugh; FFI, Fried Frailty Index; LFI, Liver Frailty Index; MELD, Model for End-Stage Liver Disease; ML, machine learning; MVR, mitral valve replacement; RCT, randomized controlled trial; ROTEM, rotational thromboelastometry; TAVR, transcatheter aortic valve replacement; TEER, transcatheter edge-to-edge repair; TEG, thromboelastography; TIPS, transjugular intrahepatic portosystemic shunt.

Off-pump vs. on-pump CABG: While observational data suggest a mortality reduction with off-pump CABG in cirrhotic cohorts (approximately 0%–5% vs. 10%–30% for on-pump CABG), these findings are derived from small retrospective series with significant selection bias [5,40]. No randomized controlled trials have been conducted. Thus, critical unanswered questions include whether the apparent superiority of off-pump CABG persists across all CTP and MELD categories or is limited to patients with CTP A disease or low MELD scores. It also remains unclear whether specific coronary anatomies, such as diffuse disease, small targets, or intramyocardial vessels, are unsuitable for off-pump approaches in cirrhosis, where incomplete revascularization may be especially harmful. Additional uncertainties include the impact of incomplete revascularization, which is more common with off-pump techniques, on long-term outcomes in a population with reduced life expectancy, and whether surgeon experience influences outcomes enough to justify restricting off-pump CABG in cirrhotic patients to high-volume centers.

Optimal MELD threshold for surgical contraindication: Published studies have variably identified MELD scores >18, >15, or >13.5 as “prohibitive” for cardiac surgery [4,5,15]. This lack of consensus reflects heterogeneous surgical populations, differences in procedure type (CABG vs. valve surgery vs. combined procedures), and evolving surgical and anesthetic techniques. The optimal MELD cutoff likely varies by procedure type; for example, a MELD score >15 may be acceptable for isolated CABG but prohibitive for valve replacement, which typically requires longer CPB times. Important unresolved questions include whether surgical center experience and volume modify acceptable MELD thresholds and whether MELD thresholds should be dynamic rather than fixed, adjusted for procedure complexity, urgency, and the availability of transcatheter alternatives.

Portal decompression timing: Whether transjugular intrahepatic portosystemic shunt (TIPS) should be performed before cardiac surgery in patients with severe portal hypertension (hepatic venous pressure gradient (HVPG) >20 mmHg) to reduce perioperative bleeding risk and ascites remains uncertain. Limited case series suggest possible benefit; however, TIPS carries its own mortality risk (~5–10%), may worsen cardiac function by increasing venous return and preload, and may increase the risk of infectious endocarditis in patients subsequently undergoing valvular procedures [19,20,21]. The optimal timing of TIPS preoperative vs. staged procedures, the appropriate patient selection criteria, including the HVPG threshold that would justify intervention, and the subsequent impact on long-term outcomes all remain undefined. Prospective comparative studies are urgently needed.

Transcatheter vs. surgical valve replacement: TAVI and TEER have expanded dramatically, offering the advantage of avoiding CPB. However, comparative outcomes data for TAVI versus surgical aortic valve replacement (SAVR) specifically in cirrhotic populations remain extremely limited. Several important questions remain unanswered: Do differences in durability between bioprosthetic surgical valves and transcatheter valves matter in a population with reduced life expectancy? Are paravalvular leak rates higher with TAVI in cirrhotic patients with coagulopathy? What are the optimal post-TAVI anticoagulation strategies in cirrhosis? Cost-effectiveness analyses comparing transcatheter and surgical approaches in cirrhotic populations are also lacking. Registry data from transcatheter valve programs should stratify outcomes by liver disease severity to inform decision-making.

Anticoagulation paradox: Cirrhotic patients exhibit a “rebalanced” hemostatic state characterized by simultaneous bleeding and thrombotic risk [7]. Optimal perioperative anticoagulation strategies remain poorly studied. Thus, key questions remain: Should VTE prophylaxis be administered at standard or reduced doses in cirrhosis? What are the appropriate prosthetic valve anticoagulation INR targets (standard 2.5–3.5 for mitral vs. lower 2–2.5)? Are DOACs truly contraindicated in mild cirrhosis (CTP A), or is this assumption based primarily on pharmacokinetic concerns rather than clinical outcomes data? The recent availability of DOAC reversal agents may change risk-benefit calculations. Prospective studies of anticoagulation strategies specifically in cirrhotic patients undergoing cardiac surgery are needed.

Machine learning and cardiac surgery-specific risk models: As discussed, VOCAL-Penn represents an advance over traditional scores but was derived from general surgical populations rather than for patients undergoing cardiac surgery specifically [19]. Currently, no cardiac surgery-specific machine learning model exists for cirrhotic patients. Development of such models will require: (1) prospective, multicenter cardiac surgery databases in cirrhotic populations with standardized data collection; (2) inclusion of cardiac surgery-specific variables, such as CPB duration, aortic cross-clamp time, procedure type, on-pump vs. off-pump approaches, and transcatheter vs. surgical interventions; (3) integration of advanced cardiac imaging parameters, including E/e' ratio, TAPSE, right ventricle (RV) strain, IVC collapsibility; (4) incorporation of functional and frailty metrics; (5) application of machine learning techniques, such as gradient boosting and neural networks, to capture complex non-linear interactions between hepatic and cardiac parameters. This represents a high-priority research direction that could substantially improve preoperative risk stratification and patient selection.

Long-term outcomes data: Most published studies report 30-day or in-hospital mortality. Data on long-term (>5-year) survival, quality of life, hepatic disease progression, and the subsequent need for liver transplantation remain extremely limited. Do cirrhotic patients who survive cardiac surgery experience accelerated hepatic decompensation? What proportion become transplant candidates within 1–5 years? Does cardiac surgery “unmask” previously compensated cirrhosis and accelerate progression to ACLF? Long-term follow-up registries are needed to answer these questions and inform shared decision-making.

#### 2.5.8 Frailty Assessment and Functional Reserve

In addition to traditional liver severity scores (CTP, MELD) and emerging surgical risk models (VOCAL-Penn, Mayo), there is growing recognition that frailty indices may provide complementary prognostic information in cirrhotic surgical candidates. Frailty, defined as a state of reduced physiological reserve and increased vulnerability to stressors, has been shown to independently predict mortality, complications, and readmission in cirrhotic populations. The Liver Frailty Index (LFI), which incorporates objective physical performance measures such as grip strength, chair stands, and balance testing, has demonstrated prognostic value in cirrhotic patients awaiting liver transplantation and undergoing general surgical procedures. Frail patients with cirrhosis experience significantly higher rates of postoperative complications, prolonged hospitalization, and mortality compared to associated non-frail counterparts, even after controlling for MELD score [61]. In the context of cardiac surgery, frailty assessment tools such as the Fried Frailty Index (FFI), Clinical Frailty Scale (CFS), and LFI may capture dimensions of patient vulnerability, including sarcopenia, functional decline, and reduced cardiopulmonary reserve, that are not reflected in biochemical scoring systems alone. Preliminary data from Jenkins et al. [22] suggest that adding frailty assessment to VOCAL-Penn may modestly improve discrimination for postoperative mortality. However, no studies have systematically evaluated the incremental predictive value of frailty indices specifically in cirrhotic patients undergoing cardiac surgery [22]. Key unanswered questions include: Which frailty assessment tool (LFI, FFI, CFS, or others) is most predictive in this population? Does frailty assessment improve risk stratification beyond VOCAL-Penn and MELD-Na? Can preoperative prehabilitation interventions targeting frailty components, such as nutrition, exercise, and functional training, reduce perioperative risk in frail cirrhotic patients? Integration of frailty metrics into preoperative assessment represents an important future direction that could enhance patient selection and guide targeted interventions.

The seven major controversies discussed above are off-pump versus on-pump CABG, optimal MELD thresholds, portal decompression timing, transcatheter versus surgical interventions, anticoagulation management, machine learning models, and frailty assessment are systematically organized in Table 2, which outlines the proposed study designs and research methodologies needed to address each area of uncertainty.

#### 2.5.9 Knowledge Gaps Requiring Research

- No prospective randomized trials have evaluated surgical techniques (on-pump vs. off-pump; conventional vs. minimally invasive) specifically in patients with cirrhosis.

- No validated frailty assessment tools exist for cirrhotic candidates undergoing cardiac surgery.

- The role of perioperative liver support devices (e.g., MARS, Prometheus) in high-risk cases remains undefined.

- No evidence-based ICU monitoring protocols (frequency of laboratory testing, hemodynamic targets, and weaning strategies) have been developed specifically for cirrhotic patients undergoing cardiac surgery.

- Data remain limited regarding the impact of specific cardiac procedures (CABG vs. aortic valve replacement (AVR) vs. mitral valve replacement (MVR) vs. combined procedures) on hepatic outcomes.

- No studies have examined the optimal timing of cardiac surgery in relation to episodes of hepatic decompensation (e.g., how long after variceal bleeding, ascites development, or encephalopathy is it safe to proceed?).

### 2.6 Limitations

This narrative review has several important limitations. First, as a non-systematic review, this review did not employ a formal systematic search strategy with predefined inclusion and exclusion criteria, which may have introduced selection bias into the included literature. In addition, no protocol was registered, and no formal risk-of-bias assessments were performed for included studies. Second, the evidence base for the perioperative management of cirrhotic patients undergoing cardiac surgery is derived predominantly from retrospective observational studies, small case series, and expert opinion rather than high-quality randomized controlled trials (see Table 2 for evidence grading). The scarcity of Level A evidence reflects the challenges of conducting rigorous prospective research in this high-risk, relatively uncommon population. Accordingly, many recommendations in this review are based on Level B (observational studies) or Level C (expert consensus) evidence. Third, significant heterogeneity exists across published studies with respect to definitions of cirrhosis severity (including varying CTP and MELD thresholds), surgical procedure types (CABG vs. valve vs. combined), CPB strategy (on-pump vs. off-pump), and outcome reporting (30-day vs. in-hospital vs. 90-day mortality), which limits our ability to provide precise and universally applicable recommendations. Fourth, many cited references address general surgical populations or broader cirrhotic cohorts rather than patients undergoing cardiac surgery specifically, necessitating cautious extrapolation. Where cardiac surgery-specific data were unavailable, the review relied on general surgical or hepatology literature, with this clearly noted. Fifth, rapidly evolving transcatheter technologies (TAVI, TEER, transcatheter tricuspid repair) and novel machine learning-based risk prediction tools (VOCAL-Penn and emerging artificial intelligence (AI) models) mean that some recommendations may require revision as additional evidence becomes available. Although the VOCAL-Penn score has demonstrated superior discrimination in mixed surgical cohorts, this metric was derived from a predominantly male veteran population and requires validation in women, non-Western populations, and exclusively cardiac surgical cohorts before the superiority of the model can be definitively established in these specific contexts. The field is dynamic, and guidelines should be revisited periodically. Sixth, the generalizability of recommendations derived predominantly from Western populations (North America and Europe) to global cardiac surgical practice remains uncertain, as cirrhosis etiologies (viral vs. alcohol-related vs. nonalcoholic steatohepatitis (NASH)), healthcare infrastructure, and access to transplantation vary globally. Finally, we acknowledge that potential publication bias, negative results, and unsuccessful outcomes in cirrhotic cardiac surgery may be underreported, leading to overly optimistic impressions of feasibility and safety. Despite these limitations, we believe this review synthesizes the best available evidence and provides practical guidance for clinicians managing this challenging population. The strength of evidence supporting each major recommendation is systematically summarized in Table 3 (Ref. [2,3,4,5,7,8,12,13,15,16,19,20,21,25,26,28,29,40,41,42,45,50,51,52]), which provides a transparent assessment of the quality of the supporting data. As shown in Table 2, many recommendations are based on Level B (observational studies) or Level C (expert opinion) evidence, reflecting the predominance of retrospective cohorts and the lack of randomized controlled trials in this high-risk population.

**Table 3.** **Evidence grading summary for key recommendations**.

Recommendation	Evidence level	Supporting evidence
Combined risk assessment (cardiac + hepatic scores).	Level B	Multiple observational cohorts have shown that EuroSCORE/STS underestimates risk [2,3,4,5].
MELD scores >18–20 represent a prohibitive surgical risk.	Level B	Supported by consistent retrospective cohort data [4,5,15].
VOCAL-Penn score is superior to MELD/Mayo.	Level B	Derivation/validation cohorts (C-statistic 0.859 vs. 0.766 vs. 0.752), with external validation [19].
Mayo score for surgical risk prediction.	Level B	Large validation studies in cirrhotic surgical patients may overestimate contemporary risk [20].
Off-pump CABG is preferred over on-pump CABG in patients with cirrhosis.	Level B	Retrospective cohort studies have shown a reduction in mortality (0–5% vs. 10–30%) [5,40].
CPB duration should be minimized (<100–120 min).	Level B	Observational data link prolonged CPB to worse outcomes [8,12].
Albumin-containing CPB prime is preferred.	Level B	Supported by non-randomized studies and meta-analyses [41].
Tranexamic acid reduces bleeding.	Level A	Evidence from an RCT [42].
Viscoelastic testing is superior to conventional laboratory tests.	Level B	RCT in cirrhosis, observational data [28,29].
Restrictive transfusion strategy (Hgb 8–9 g/dL).	Level A	RCT evidence from general populations [45].
INR 1.5–1.8 should not be routinely corrected.	Level B	Cohort studies, expert consensus [7,28].
Early CVVH for AKI improves outcomes.	Level A	Multiple RCTs (STARRT-AKI, Gaudry) [50,51,52].
Heart–Liver Team multidisciplinary evaluation.	Level C	Expert consensus, no controlled trials [5,16,19].
Screening for cirrhotic cardiomyopathy (E/e' ratio).	Level C	Expert opinion, pathophysiological rationale [25,26].
Prophylactic variceal therapy before surgery.	Level B	Cohort data, guideline recommendations [21].
TAVI/TEER is preferred over surgery in high-risk cirrhosis.	Level C	Case series, expert consensus, lack of comparative data [19].
CTP C is a relative contraindication to cardiac surgery.	Level B	Consistent observational data has shown ~70% mortality [5,12,13].
Cardiac surgery-specific ML models.	Level C	Research gap: no validated cardiac surgery-specific ML models exist; this remains a high-priority future direction.

Evidence levels: Level A, high quality (RCTs, systematic reviews, and large prospective cohorts with consistent findings); Level B, moderate quality (non-randomized studies, retrospective cohorts, and observational studies); Level C, low quality (expert opinion, case series, small retrospective studies, and guideline consensus). Abbreviations: AKI, acute kidney injury; CABG, coronary artery bypass grafting; CPB, cardiopulmonary bypass; CTP, Child–Turcotte–Pugh; CVVH, continuous veno-venous hemofiltration; E/e', ratio of early mitral inflow velocity to early diastolic mitral annular velocity; EuroSCORE, European System for Cardiac Operative Risk Evaluation; Hgb, hemoglobin; INR, international normalized ratio; MELD, Model for End-Stage Liver Disease; ML, machine learning; RCT, randomized controlled trial; STARRT-AKI, Standard versus Accelerated Initiation of Renal Replacement Therapy in Acute Kidney Injury (trial); STS, Society of Thoracic Surgeons (risk score); TAVI, transcatheter aortic valve implantation; TEER, transcatheter edge-to-edge repair; VOCAL-Penn, Veterans Outcomes and Costs Associated with Liver Disease – Penn (surgical risk score).

## 3. Conclusions

Liver cirrhosis remains a major, and often under-recognized, modifier of perioperative risk in cardiac surgery, conferring a 4–7-fold increase in mortality and morbidity [4,5,12]. The unique pathophysiological alterations associated with cirrhosis, such as hemodynamic dysfunction, fragile coagulation, renal vulnerability, and immune dysregulation, interact synergistically with surgical stress to produce exceptionally high-risk clinical scenarios [7,10,13,16].

Thus, integrating liver-specific risk assessment tools (CTP, MELD, and VOCAL-Penn or Mayo scores) into preoperative evaluation, considering emerging machine learning predictive tools as these models become validated for cardiac surgery populations, mandating multidisciplinary Heart–Liver Team discussions, systematically optimizing modifiable factors before surgery (ascites, portal hypertension, coagulopathy, encephalopathy, infection, and malnutrition), applying evidence-based intraoperative strategies (such as minimizing CPB duration, favoring off-pump approaches when feasible, using viscoelastic-guided transfusion, and ensuring meticulous hemodynamic management), and providing vigilant postoperative care in a specialized ICU can meaningfully improve outcomes in carefully selected cirrhotic patients undergoing cardiac surgery [5,12,16,19,28,35,39].

Critical knowledge gaps remain regarding optimal surgical techniques, anticoagulation strategies, the timing of portal decompression, and long-term outcomes. The development of cardiac surgery-specific machine learning risk prediction models and prospective multicenter registries represents a high-priority research direction. By applying the phase-oriented approach outlined in this review, with emphasis on considerations before, during, and after surgery, cardiac surgeons can optimize outcomes and reduce preventable complications in this challenging population [16,17,18,19,20,35,39].

## References

[1] GBD 2017 Cirrhosis Collaborators (2020). The global, regional, and national burden of cirrhosis by cause in 195 countries and territories, 1990-2017: a systematic analysis for the Global Burden of Disease Study 2017. The Lancet. Gastroenterology & Hepatology.

[2] Nashef SAM, Roques F, Sharples LD, Nilsson J, Smith C, Goldstone AR (2012). EuroSCORE II. European Journal of Cardio-thoracic Surgery : Official Journal of the European Association for Cardio-thoracic Surgery.

[3] Shahian DM, Jacobs JP, Badhwar V, Kurlansky PA, Furnary AP, Cleveland JC (2018). The Society of Thoracic Surgeons 2018 Adult Cardiac Surgery Risk Models: Part 1-Background, Design Considerations, and Model Development. The Annals of Thoracic Surgery.

[4] Suman A, Barnes DS, Zein NN, Levinthal GN, Connor JT, Carey WD (2004). Predicting outcome after cardiac surgery in patients with cirrhosis: a comparison of Child-Pugh and MELD scores. Clinical Gastroenterology and Hepatology : the Official Clinical Practice Journal of the American Gastroenterological Association.

[5] Garatti A, Daprati A, Cottini M, Russo CF, Dalla Tomba M, Troise G (2021). Cardiac Surgery in Patients With Liver Cirrhosis (CASTER) Study: Early and Long-Term Outcomes. The Annals of Thoracic Surgery.

[6] Møller S, Henriksen JH (2010). Cirrhotic cardiomyopathy. Journal of Hepatology.

[7] Tripodi A, Mannucci PM (2011). The coagulopathy of chronic liver disease. The New England Journal of Medicine.

[8] Paparella D, Yau TM, Young E (2002). Cardiopulmonary bypass induced inflammation: pathophysiology and treatment. An update. European Journal of Cardio-thoracic Surgery : Official Journal of the European Association for Cardio-thoracic Surgery.

[9] Kamath PS, Wiesner RH, Malinchoc M, Kremers W, Therneau TM, Kosberg CL (2001). A model to predict survival in patients with end-stage liver disease. Hepatology (Baltimore, Md.).

[10] Angeli P, Gines P, Wong F, Bernardi M, Boyer TD, Gerbes A (2015). Diagnosis and management of acute kidney injury in patients with cirrhosis: revised consensus recommendations of the International Club of Ascites. Gut.

[11] Biggins SW, Kim WR, Terrault NA, Saab S, Balan V, Schiano T (2006). Evidence-based incorporation of serum sodium concentration into MELD. Gastroenterology.

[12] Jacob KA, Hjortnaes J, Kranenburg G, de Heer F, Kluin J (2015). Mortality after cardiac surgery in patients with liver cirrhosis classified by the Child-Pugh score. Interactive Cardiovascular and Thoracic Surgery.

[13] Moreau R, Jalan R, Gines P, Pavesi M, Angeli P, Cordoba J (2013). Acute-on-chronic liver failure is a distinct syndrome that develops in patients with acute decompensation of cirrhosis. Gastroenterology.

[14] Shimamura J, Okumura K, Misawa R, Bodin R, Nishida S, Tavolacci S (2024). Strategy and Outcomes of Cardiac Surgery in Patients With Cirrhosis: Comprehensive Approach With Liver Transplant Program. Clinical Transplantation.

[15] Hawkins RB, Young BAC, Mehaffey JH, Speir AM, Quader MA, Rich JB (2019). Model for End-Stage Liver Disease Score Independently Predicts Mortality in Cardiac Surgery. The Annals of Thoracic Surgery.

[16] European Association for the Study of the Liver (2018). EASL Clinical Practice Guidelines for the management of patients with decompensated cirrhosis. Journal of Hepatology.

[17] European Association for the Study of the Liver (2025). EASL Clinical Practice Guidelines on extrahepatic abdominal surgery in patients with cirrhosis and advanced chronic liver disease. Journal of Hepatology.

[18] Gerlach RM (2024). Cirrhosis: Getting to the Heart of the Matter. Journal of Cardiothoracic and Vascular Anesthesia.

[19] Mahmud N, Fricker Z, Hubbard RA, Ioannou GN, Lewis JD, Taddei TH (2021). Risk Prediction Models for Post-Operative Mortality in Patients With Cirrhosis. Hepatology (Baltimore, Md.).

[20] Teh SH, Nagorney DM, Stevens SR, Offord KP, Therneau TM, Plevak DJ (2007). Risk factors for mortality after surgery in patients with cirrhosis. Gastroenterology.

[21] Jadaun SS, Saigal S (2022). Surgical Risk Assessment in Patients with Chronic Liver Diseases. Journal of Clinical and Experimental Hepatology.

[22] Jenkins H, Awad AK, Poddi S, Rosinski B, O'Shea R, Xu B (2026). Validation of current mortality risk prediction models in patients with cirrhosis undergoing cardiac surgery. JTCVS Open.

[23] Northup PG, Garcia-Pagan JC, Garcia-Tsao G, Intagliata NM, Superina RA, Roberts LN (2021). Vascular Liver Disorders, Portal Vein Thrombosis, and Procedural Bleeding in Patients With Liver Disease: 2020 Practice Guidance by the American Association for the Study of Liver Diseases. Hepatology (Baltimore, Md.).

[24] European Association for the Study of the Liver (2018). EASL Clinical Practice Guidelines: Management of hepatocellular carcinoma. Journal of Hepatology.

[25] Izzy M, VanWagner LB, Lin G, Altieri M, Findlay JY, Oh JK (2020). Redefining Cirrhotic Cardiomyopathy for the Modern Era. Hepatology (Baltimore, Md.).

[26] Nagueh SF, Smiseth OA, Appleton CP, Byrd BF, Dokainish H, Edvardsen T (2016). Recommendations for the Evaluation of Left Ventricular Diastolic Function by Echocardiography: An Update from the American Society of Echocardiography and the European Association of Cardiovascular Imaging. Journal of the American Society of Echocardiography : Official Publication of the American Society of Echocardiography.

[27] Rudski LG, Lai WW, Afilalo J, Hua L, Handschumacher MD, Chandrasekaran K (2010). Guidelines for the echocardiographic assessment of the right heart in adults: a report from the American Society of Echocardiography endorsed by the European Association of Echocardiography, a registered branch of the European Society of Cardiology, and the Canadian Society of Echocardiography. Journal of the American Society of Echocardiography : Official Publication of the American Society of Echocardiography.

[28] European Association for the Study of the Liver (2022). EASL Clinical Practice Guidelines on prevention and management of bleeding and thrombosis in patients with cirrhosis. Journal of Hepatology.

[29] De Pietri L, Bianchini M, Montalti R, De Maria N, Di Maira T, Begliomini B (2016). Thrombelastography-guided blood product use before invasive procedures in cirrhosis with severe coagulopathy: A randomized, controlled trial. Hepatology (Baltimore, Md.).

[30] European Association for the Study of the Liver (2022). EASL Clinical Practice Guidelines on the management of hepatic encephalopathy. Journal of Hepatology.

[31] European Association for the Study of the Liver (2019). EASL Clinical Practice Guidelines on nutrition in chronic liver disease. Journal of Hepatology.

[32] Gilbert-Kawai N, Hogan B, Milan Z (2022). Perioperative management of patients with liver disease. BJA Education.

[33] Starczewska MH, Mon W, Shirley P (2017). Anaesthesia in patients with liver disease. Current Opinion in Anaesthesiology.

[34] Egan TD (1995). Remifentanil pharmacokinetics and pharmacodynamics. A preliminary appraisal. Clinical Pharmacokinetics.

[35] Saugel B, Fletcher N, Gan TJ, Grocott MPW, Myles PS, Sessler DI (2024). PeriOperative Quality Initiative (POQI) international consensus statement on perioperative arterial pressure management. British Journal of Anaesthesia.

[36] Nicoara A, Skubas N, Ad N, Finley A, Hahn RT, Mahmood F (2020). Guidelines for the Use of Transesophageal Echocardiography to Assist with Surgical Decision-Making in the Operating Room: A Surgery-Based Approach: From the American Society of Echocardiography in Collaboration with the Society of Cardiovascular Anesthesiologists and the Society of Thoracic Surgeons. Journal of the American Society of Echocardiography : Official Publication of the American Society of Echocardiography.

[37] Lopez MG, Shotwell MS, Morse J, Liang Y, Wanderer JP, Absi TS (2021). Intraoperative venous congestion and acute kidney injury in cardiac surgery: an observational cohort study. British Journal of Anaesthesia.

[38] Monnet X, Messina A, Greco M, Bakker J, Aissaoui N, Cecconi M (2025). ESICM guidelines on circulatory shock and hemodynamic monitoring 2025. Intensive Care Medicine.

[39] Wahba A, Kunst G, De Somer F, Agerup Kildahl H, Milne B, Kjellberg G (2025). 2024 EACTS/EACTAIC/EBCP Guidelines on cardiopulmonary bypass in adult cardiac surgery. European Journal of Cardio-thoracic Surgery : Official Journal of the European Association for Cardio-thoracic Surgery.

[40] Gopaldas RR, Chu D, Cornwell LD, Dao TK, LeMaire SA, Coselli JS (2013). Cirrhosis as a moderator of outcomes in coronary artery bypass grafting and off-pump coronary artery bypass operations: a 12-year population-based study. The Annals of Thoracic Surgery.

[41] Skubas NJ, Callum J, Bathla A, Keshavarz H, Fergusson D, Wu B (2024). Intravenous albumin in cardiac and vascular surgery: a systematic review and meta-analysis. British Journal of Anaesthesia.

[42] Myles PS, Smith JA, Forbes A, Silbert B, Jayarajah M, Painter T (2017). Tranexamic Acid in Patients Undergoing Coronary-Artery Surgery. The New England Journal of Medicine.

[43] Carless PA, Henry DA, Moxey AJ, O'Connell D, Brown T, Fergusson DA (2010). Cell salvage for minimising perioperative allogeneic blood transfusion. The Cochrane Database of Systematic Reviews.

[44] Kozek-Langenecker SA, Ahmed AB, Afshari A, Albaladejo P, Aldecoa C, Barauskas G (2017). Management of severe perioperative bleeding: guidelines from the European Society of Anaesthesiology: First update 2016. European Journal of Anaesthesiology.

[45] Carson JL, Stanworth SJ, Roubinian N, Fergusson DA, Triulzi D, Doree C (2016). Transfusion thresholds and other strategies for guiding allogeneic red blood cell transfusion. The Cochrane Database of Systematic Reviews.

[46] Biggins SW, Angeli P, Garcia-Tsao G, Ginès P, Ling SC, Nadim MK (2021). Diagnosis, Evaluation, and Management of Ascites, Spontaneous Bacterial Peritonitis and Hepatorenal Syndrome: 2021 Practice Guidance by the American Association for the Study of Liver Diseases. Hepatology (Baltimore, Md.).

[47] Arabi YM, Belley-Cote E, Carsetti A, De Backer D, Donadello K, Juffermans NP (2024). European Society of Intensive Care Medicine clinical practice guideline on fluid therapy in adult critically ill patients. Part 1: the choice of resuscitation fluids. Intensive Care Medicine.

[48] Mekontso Dessap A, AlShamsi F, Belletti A, De Backer D, Delaney A, Møller MH (2025). European Society of Intensive Care Medicine (ESICM) 2025 clinical practice guideline on fluid therapy in adult critically ill patients: part 2-the volume of resuscitation fluids. Intensive Care Medicine.

[49] Ostermann M, Auzinger G, Grocott M, Morton-Bailey V, Raphael J, Shaw AD (2024). Perioperative fluid management: evidence-based consensus recommendations from the international multidisciplinary PeriOperative Quality Initiative. British Journal of Anaesthesia.

[50] Gaudry S, Hajage D, Schortgen F, Martin-Lefevre L, Pons B, Boulet E (2016). Initiation Strategies for Renal-Replacement Therapy in the Intensive Care Unit. The New England Journal of Medicine.

[51] STARRT-AKI Investigators, Canadian Critical Care Trials Group, Australian and New Zealand Intensive Care Society Clinical Trials Group, United Kingdom Critical Care Research Group, Canadian Nephrology Trials Network, Irish Critical Care Trials Group (2020). Timing of Initiation of Renal-Replacement Therapy in Acute Kidney Injury. The New England Journal of Medicine.

[52] Gaudry S, Hajage D, Benichou N, Chaïbi K, Barbar S, Zarbock A (2020). Delayed versus early initiation of renal replacement therapy for severe acute kidney injury: a systematic review and individual patient data meta-analysis of randomised clinical trials. Lancet (London, England).

[53] Klompas M, Branson R, Cawcutt K, Crist M, Eichenwald EC, Greene LR (2022). Strategies to prevent ventilator-associated pneumonia, ventilator-associated events, and nonventilator hospital-acquired pneumonia in acute-care hospitals: 2022 Update. Infection Control and Hospital Epidemiology.

[54] Buetti N, Marschall J, Drees M, Fakih MG, Hadaway L, Maragakis LL (2022). Strategies to prevent central line-associated bloodstream infections in acute-care hospitals: 2022 Update. Infection Control and Hospital Epidemiology.

[55] Bratzler DW, Dellinger EP, Olsen KM, Perl TM, Auwaerter PG, Bolon MK (2013). Clinical practice guidelines for antimicrobial prophylaxis in surgery. American Journal of Health-system Pharmacy : AJHP : Official Journal of the American Society of Health-System Pharmacists.

[56] Roberts LN, Hernandez-Gea V, Magnusson M, Stanworth S, Thachil J, Tripodi A (2022). Thromboprophylaxis for venous thromboembolism prevention in hospitalized patients with cirrhosis: Guidance from the SSC of the ISTH. Journal of Thrombosis and Haemostasis: JTH.

[57] Van Gelder IC, Rienstra M, Bunting KV, Casado-Arroyo R, Caso V, Crijns HJGM (2024). 2024 ESC Guidelines for the management of atrial fibrillation developed in collaboration with the European Association for Cardio-Thoracic Surgery (EACTS). European Heart Journal.

[58] Schaller SJ, Scheffenbichler FT, Bein T, Blobner M, Grunow JJ, Hamsen U (2024). Guideline on positioning and early mobilisation in the critically ill by an expert panel. Intensive Care Medicine.

[59] Mertes PM, Kindo M, Amour J, Baufreton C, Camilleri L, Caus T (2022). Guidelines on enhanced recovery after cardiac surgery under cardiopulmonary bypass or off-pump. Anaesthesia, Critical Care & Pain Medicine.

[60] Plauth M, Bernal W, Dasarathy S, Merli M, Plank LD, Schütz T (2019). ESPEN guideline on clinical nutrition in liver disease. Clinical Nutrition (Edinburgh, Scotland).

[61] Olson SL, Polineni P, Schwartz WAH, Thuluvath AJ, Duarte-Rojo A, Ladner DP (2024). Comparing Functional Frailty and Radiographic Sarcopenia as Predictors of Outcomes After Liver Transplant. Clinical Transplantation.

